# Radiotherapy quality assurance in the PRO-GLIO trial: results from a dummy run comparing experts across twelve institutions in two Scandinavian countries

**DOI:** 10.1016/j.ctro.2026.101220

**Published:** 2026-06-18

**Authors:** Liv Cathrine Heggebø, Taran Paulsen Hellebust, Thomas Henry, Lars Fredrik Fjæra, Andreas Ottestad, Hillevi Rylander, Maziar Hervani, Morten Egeberg Evensen, Magnus Gustafsson, Hanne Blakstad, Mette Sprauten, Henriette Magelssen, Janani Moorthy, Karin M. Andersson, Christina Vallhagen Dahlgren, Dorota Goplen, Jorunn Brekke, Tor-Christian Aase Johannessen, Tora S. Solheim, Kirsten Marienhagen, Per Bergström, Måns Agrup, Sara Kinhult, Sara Alkner, Ludvig Dahl, Ingrid Kristensen, Michael Gubanski, Mark Zupancic, Michael Strandeus, Ingela Raunert, Anna Flejmer, Frida Jakobsson, Christina Ramberg, Katja Werlenius, Malin Blomstrand, Petter Brandal

**Affiliations:** a Department of Oncology, Oslo University Hospital, Oslo, Norway; b Institute for Clinical Medicine, University of Oslo, Oslo, Norway; c Department of Medical Physics, Oslo University Hospital, Oslo, Norway; d Faculty of Health and Social Sciences, University of South-Eastern Norway, Norway; e Department of Medical Physics and Biomedical Engineering, Sahlgrenska University Hospital, Gothenburg, Sweden; f Department of Medical Radiation Science, Institute of Clinical Sciences, Sahlgrenska Academy, University of Gothenburg, Gothenburg, Sweden; g The Skandion Clinic, Uppsala, Sweden; h Section of Oncology, Drammen Hospital, Vestre Viken Hospital Trust, Drammen, Norway; i Department of Immunology, Genetics and Pathology, Uppsala University, Uppsala, Sweden; j Department of Oncology and Medical Physics, Haukeland University Hospital, Bergen, Norway; k Cancer Clinic, St. Olavs Hospital, Trondheim University Hospital, Trondheim, Norway; l Department of Clinical and Molecular Medicine, Faculty of Medicine and Health Sciences, Norwegian University of Science and Technology, Trondheim, Norway; m Department of Oncology, University Hospital of North Norway, Tromsø, Norway; n Department of Oncology, Northern Sweden University Hospital, Umea, Sweden; o Department of Oncology, Linköping University Hospital, Linköping, Sweden; p Department of Biomedical and Clinical Sciences, Linköping University, Linköping, Sweden.; q Department of Hematology, Oncology and Radiation Physics, Skåne University Hospital, Lund, Sweden; r Department of Oncology, Institution of Clinical Sciences Lund, Lund University, Sweden; s Department of Radiotherapy, Karolinska University Hospital, Stockholm, Sweden; t Medical Unit Head, Neck, Lung, and Skin Cancer, Theme Cancer, Karolinska University Hospital, Stockholm, Sweden; u Department of Oncology, The Ryhov County Hospital, Jönköping, Sweden; v Uppsala University Hospital, Uppsala, Sweden; w Department of Oncology, Örebro University Hospital, Örebro, Sweden; x Department of Oncology, Sahlgrenska University Hospital, Gothenburg, Sweden; y Department of Oncology, Institute of Clinical Sciences, Sahlgrenska Academy, University of Gothenburg, Gothenburg, Sweden

**Keywords:** Quality assurance, *IDH*-mutated glioma, Interobserver variability, Deep learning segmentation

## Abstract

**Background:**

Target volume and organ of interest (OOI) contouring and treatment planning for *IDH*-mutated gliomas CNS WHO grades 2 and 3 are subject to interindividual variation. We aimed to assess this variation and heighten delineation awareness as a quality assurance measure in the PRO-GLIO trial.

**Methods:**

Five experts established consensus delineations for target volumes and defined OOI in two *IDH*-mutated gliomas cases. Next, target volumes and OOI were delineated by experts from 12 treatment centers participating in the PRO-GLIO trial, and by deep learning segmentation (DLS). These structures were compared to consensus contours based on predefined quantitative and qualitative parameters. Proton and photon treatment planning were performed on the consensus delineations by 11 centers.

**Results:**

Median dice similarity coefficient (DSC) for clinical target volume (CTV) was 0.92 for both cases, whereas the median 95th percentile Hausdorff distance for CTV was 0.54 cm and 0.65 cm. Larger variability in DSC and volume size was seen for OOI, e.g., a median DSC of 0.47 and 0.64 for the optic chiasm and between 0.61 and 0.77 for the hippocampi. DSC-derived DLS-values were within the range of manual delineations for all OOI.

**Conclusions:**

Delineation variation between study centers was minor for target volumes, especially for DSC of CTV. Variability was considerably higher for several OOI, representing a potential hazard not least with new and more precise radiotherapy techniques. Interestingly, DLS performed OOI-segmentation within the range of manual experts and represents a reasonable OOI-segmentation alternative in the future.

## Highlights

• PRO-GLIO dummy run procedure assessed contouring variation across 12 centers.
• Consistency in target volume delineation across study centers.
• Considerable contouring variability for several organs of interest across centers.
• Deep learning segmentation demonstrated potential for organ of interest delineation.

## Graphical abstract

**Figure:**
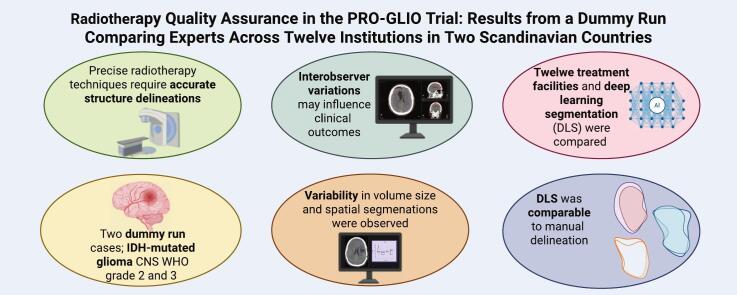

## 1 Introduction

Precision in radiotherapy deliverance has increased continuously in the latest decades, making accurate target volume and organ of interest (OOI) delineation increasingly critical for optimal treatment effect without inflicting unnecessary harm to patients [1], [2], [3], [4]. Reflecting this, quality assurance (QA) is advised in radiotherapy trials [1], [2], [4], [5], [6], and The European Organization for Research and Treatment of Cancer (EORTC) Radiation Oncology Group (ROG) has initiated a QA program in radiotherapy (QART) to ensure safe and effective treatment delivery in study settings [1], [7]. Dummy run (DR) procedures allow evaluation of structure delineation, treatment planning and protocol adherence, and enable study teams to handle systematic errors in clinical studies [2], [8], [9].

Delineation of target volumes in isocitrate dehydrogenase (*IDH*)-mutated gliomas is inherently difficult, particularly because of the diffuse tumor infiltration. In Norway and Sweden, all 12 treatment centers responsible for radiotherapy of *IDH*-mutated gliomas CNS WHO grade 2 and 3 partake in the ongoing phase III PRO-GLIO trial (NCT05190172). PRO-GLIO aims to investigate if proton beam therapy (PBT) is advantageous compared to photon therapy for this patient population [10], and patients are randomized 1:1 to radiotherapy delivered with protons or photons. Even though Norway and Sweden are small countries and fairly alike when it comes to treatment practice, interobserver variabilities in target and OOI definition exist. In multicenter clinical trials treatment planning variations pose a threat to the internal validity and generalizability of study findings [11], [12]. We therefore set out to investigate variability across the 12 study centers using a DR procedure. The purpose was to evaluate interobserver variability and ensure protocol adherence, leading to a harmonized practice and heightened quality of delineation and radiotherapy planning across study centers. Furthermore, deep learning segmentation (DLS) has emerged as a promising strategy to improve delineation consistency and reduce resource spending in radiotherapy [13], [14], [15], [16], [17], [18], [19], [20]. As a supplementary undertaking we therefore compared a DLS-based contouring tool with delineations from the study centers to evaluate the performance of the DLS-based tool.

## 2 Materials and methods

Before the 12 study centers participating in the PRO-GLIO trial (Supplementary Table 1) could commence patient inclusion, one mandatory DR case (case 1) had to be completed. This included delineation of target volumes and OOI by at least one experienced neuro-oncologist, and radiotherapy planning on consensus delineations. Consensus delineations for target volumes and OOI were established by an expert group consisting of three experienced neuro-oncologists from Oslo University Hospital (OUS) and two from Sahlgrenska University Hospital. All five expert group participants independently delineated target volumes and OOI on two DR cases. All delineations for each DR case were then imported into the same planning CT and each contour was discussed slice-by-slice in a consensus meeting until consensus delineations were reached.

All study centers made a photon plan, whereas study centers responsible for making proton plans in the study also had to provide a proton plan. The latter was optional for other study centers. Delineation and planning for the second DR case were also optional. All centers received written feedback and were offered digital feedback.

### 2.1 Dummy run case 1

Dummy run case 1 was a patient re-resected for a left-sided frontotemporal *IDH*-mutated astrocytoma CNS WHO grade 2. The only antineoplastic treatment administered before re-resection was surgery.

### 2.2 Dummy run case 2

The second patient had undergone a subtotal resection for a radiologically suspected widespread glioma. Only minor areas showed contrast enhancement, and all these were removed. Histopathology revealed an *IDH*-mutated oligodendroglioma CNS WHO grade 3.

## 3 Procedure for and evaluation of structure delineation

Target volume and OOI delineation had to be performed according to the PRO-GLIO protocol. Protocol guidelines are based on the European Particle Therapy Network (EPTN) consensus-based atlas, European Society for Radiotherapy and Oncology (ESTRO) and European Association of Neuro-Oncology (EANO) guidelines, and national Norwegian and Swedish guidelines [10], [21], [22], [23], [24]. Several OOI were mandatory, whereas others were optional (Supplementary Table 2). The periventricular zone (PVZ) was added as a mandatory OOI at a later stage and, hence, not included in treatment planning evaluation. The treatment planning system RayStation® version 2024 A SP1 was used to generate DLS segmentations for available OOI.

**Supplementary Fig. S1:**
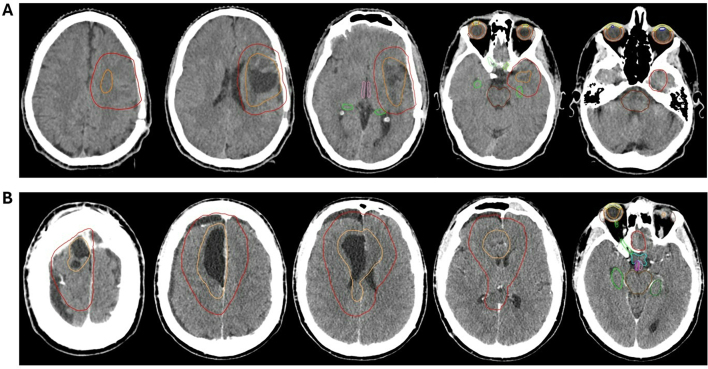
Figure displaying axial dose planning CT images including consensus delineations.

All 12 study centers had access to a short patient history including radiological descriptions, dose planning CT-scans (Supplementary Fig. 1) matched with pre- and postoperative MRI-series (Fluid-Attenuated Inversion Recovery (FLAIR) and gadolinium-enhanced T1), and an empty radiotherapy structure set. In case 1, the slice thickness of the dose planning CT-scan was 2 mm, whereas for case 2 it was 1 mm. For the first case, participants were requested to complete a brief questionnaire (Supplementary material).

**Supplementary Fig. S2:**
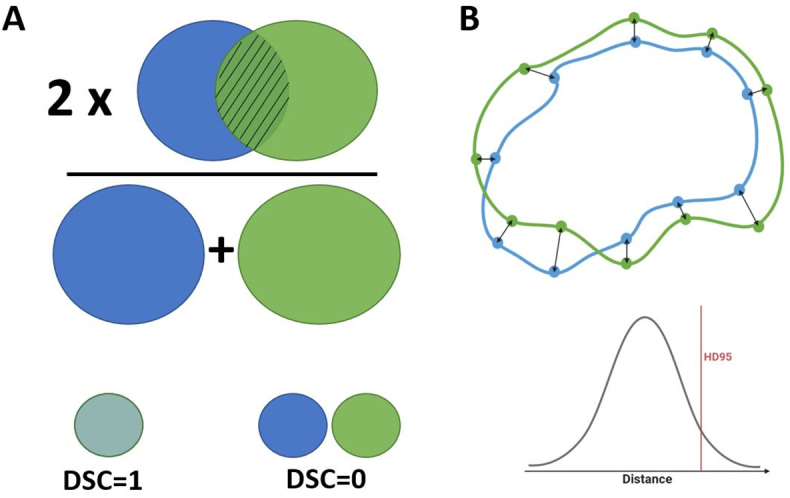
Dice similarity coefficient and 95th percentile Hausdorff distance.

A QA working group consisting of neuro-oncologists, medical physicists, and dosimetrists from OUS was responsible for evaluating delineations. Contours were evaluated qualitatively slice-by-slice based on descriptions in the PRO-GLIO-study protocol [10].DICE similarity coefficient (DSC) was chosen to evaluate size and overlap agreement between each delineated structure and the corresponding consensus delineation. DSC is defined as two times the volume where the contours overlap, divided by the total volume of the two contours combined (Supplementary Fig. 2). A DSC of 1 represents perfect agreement, whereas a value of 0 represents no overlap [25]. This definition of DSC often leads to high DSC values for large volumes, despite large interobserver variations. Therefore, the 95th percentile Hausdorff distance (HD95) was also assessed. For HD95, smaller values indicate higher agreement. The HD95 was preferred to maximum HD as HD95 eliminates the impact of outliers [26] (Supplementary Fig. 2). Structure volumes were also set against each other using ratios, e.g., the largest delineated structure volume was compared to the smallest corresponding structure volume.

## 4 Procedure for and evaluation of treatment plans

Treatment planning was performed on consensus delineations and done in accordance with the PRO-GLIO protocol [10], which is harmonized with EPTN consensus and national guidelines [27]. Radiotherapy plans were evaluated based on a set of predefined quantitative and qualitative parameters. Qualitative assessments included a visual slice-by-slice examination of the isodose distribution and evaluation of the field setup. Fulfillment of dose constraints and sparing of OOI were assessed. Also, target volume dose homogeneity, target volume dose coverage and conformity indices were evaluated (Supplementary Table 3). In addition, delineations from all study centers were imported into the same treatment plan (using plans made at OUS) using a non-replanning approach [28]. The latter was done to evaluate the potential impact of interobserver variation on target volume coverage and dose to OOI.

## 5 Results

For case 1, delineations from all 12 participating centers were evaluated, excluding the two centers that contributed to the consensus delineations. PVZ was added at a later stage and was delineated by 11 participants from 10 study centers (Table 1). In case 2, 10 delineations of the same structures stemmed from eight different study centers. The number of delineations for optional OOI varied (Table 1). DLS-based delineations for available OOI were assessed for both cases. Twelve photon plans and 9 proton plans were evaluated for case 1, and 9 photon and 7 proton plans for case 2.

**Table 1:** Number of delineators (number of study centers).

**Volume**		**Dummy run case 1**	**Dummy run case 2**
Target volumes		10 (10)*	10 (8)*
Mandatory organs of interest		10 (10)*	9 (8)*
Optional organs of intest	Corneae	6	8
	Hypothalami	4	5**
	Lacrimal glands	7	9
	Retinae	7	9
Periventricular zone***		11 (10)	NA

NA: Not applicable. *Two of 12 study centers were excluded as they contributed to the consensus delineation in case 1, whereas, two additional neurooncologists from one of the centers responsible for consensus delineations participated in case 2. **One participant delineated the hypothalami as a union not possible to separate in two adequate volumes (left and right). This one organ of interest delineation was therefore evaluated only qualitatively. ***Added at a later time point and only for case 1.

### 5.1 Volume of all delineated structures

**Fig. 1:**
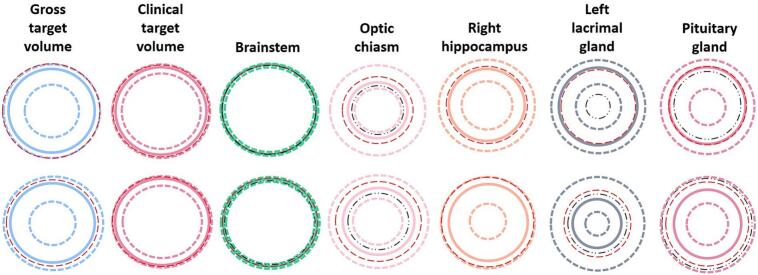
Size of delineated volumes. Upper row displays results from dummy run (DR) case 1 and lower row from DR case 2. The solid line is set as reference and represents the median volume size across study centers. The radius of the other circles is calculated as a ratio relative to the median volume. The smallest dotted circle in the same colour represents the smallest volume delineated by any study center, whereas the largest dotted circle represents the largest volume. Red dotted circle represents the volume size of the consensus delineation, and black dotted circle represents the deep learning segmentation (not available for all volumes). This means as an example that for the left lacrimal gland in case 2, the smallest volume is 16 times smaller than the largest volume, whereas the volume of the consensus delineation and DLS are larger than the median volume size across the study centers. (For interpretation of the references to colour in this figure legend, the reader is referred to the web version of this article.)

There were substantial differences in size of several delineated structure volumes across centers (Table 2, Table 3, Fig. 1), with largest variations for the left lacrimal gland in case 2 of which the largest contour was 16 times the volume of the smallest. The largest volumes for cochleae, pituitary gland and right lacrimal gland in both cases were five times bigger than the corresponding smallest. The brainstem was the structure with smallest volume variation with a range of 25.30–29.91 cm^3^ (cc) resulting in a ratio of 1.2 between the largest and smallest structure volume in case 1, and a corresponding ratio of 1.3 in case 2. DLS resulted in smaller volumes than consensus volumes for all structures except three of four cochleae, as well as the pituitary gland in case 2. Consensus delineations were larger than the median volume from the study centers for 17 and 19 of 21 structures in case 1 and 2 (Table 2, Table 3, Fig. 1). Volume size of some structures varied substantially also in the expert group, with ratios of up to four for some OOI (Supplementary Table 4).

**Table 2:** Median DSC, HD95, and volume size for all delineated structures for dummy run case 1. Target volumes and organs of interest are ranked from the largest to the smallest median volume. For all structures the consensus was used as reference.

Structure	Volume size (cm^3^) median (range)	Volume size (cm^3^) consensus	Volume size (cm^3^) DLS	DSC median (range)	DSC DLS	HD95 (cm) median (range)	HD95 (cm) DLS
CTV	194.09 (143.23–221.99)	212.20	–	0.92 (0.79–0.96)	–	0.54 (0.30–1.08)	–
GTV	59.52 (23.99–75.30)	76.54	–	0.85 (0.47–0.97)	–	0.59 (0.28–1.70)	–
Brainstem	28.37 (25.30–29.91)	27.65	27.61	0.92 (0.83–0.93)	0.92	0.27 (0.22–0.53)	0.24
Retina, right	3.22 (2.21–3.68)	3.77	–	0.60 (0.42–0.72)	–	0.31 (0.23–0.41)	–
Retina, left	3.24 (2.03–3.59)	3.37	–	0.59 (0.48–0.72)	–	0.25 (0.23–0.33)	–
Hippocampus, right	1.60 (0.71–2.58)	1.83	–	0.67 (0.50–0.78)	–	0.36 (0.27–0.63)	–
Hippocampus, left	1.99 (0.75–2.76)	1.79	–	0.61 (0.32–0.78)	–	0.43 (0.27–0.88)	–
Cornea, right	1.04 (0.77–1.55)	1.16	–	0.61 (0.30–0.73)	–	0.27 (0.24–0.35)	–
Cornea, left	1.09 (0.70–1.58)	1.14	–	0.63 (0.28–0.67)	–	0.29 (0.23–0.32)	–
Optic nerve, right	0.56 (0.38–1.02)	0.58	0.50	0.63 (0.44–0.68)	0.71	0.28 (0.22–0.41)	0.23
Optic nerve, left	0.58 (0.29–0.89)	0.64	0.44	0.51 (0.32–0.70)	0.59	0.27 (0.24–0.60)	0.25
Lacrimal gland, right	0.51 (0.16–0.95)	0.36	0.02	0.57 (0.00–0.72)	0.12	0.30 (0.21–1.48)	0.98
Lacrimal gland, left	0.53 (0.18–0.76)	0.48	0.05	0.68 (0.00–0.78)	0.20	0.23 (0.19–1.40)	0.64
Optic chiasm	0.35 (0.21–0.88)	0.49	0.26	0.47 (0.33–0.67)	0.57	0.44 (0.29–0.80)	0.36
Hypothalamus, right	0.35 (0.22–0.82)	0.52	–	0.40 (0.33–0.54)	–	0.79 (0.25–1.51)	–
Hypothalamus, left	0.38 (0.22–0.71)	0.49	–	0.52 (0.26–0.63)	–	0.73 (0.26–1.45)	–
Pituitary gland	0.27 (0.06–0.41)	0.28	0.21	0.70 (0.33–0.85)	0.55	0.25 (0.17–0.43)	0.25
Lens, right	0.22 (0.10–0.34)	0.26	0.14	0.80 (0.55–0.85)	0.70	0.18 (0.13–0.21)	0.20
Lens, left	0.21 (0.09–0.29)	0.25	0.15	0.80 (0.51–0.83)	0.73	0.18 (0.14–0.21)	0.20
Cochlea, right	0.04 (0.02–0.12)	0.13	0.16	0.49 (0.30–0.80)	0.56	0.25 (0.21–0.42)	0.38
Cochlea, left	0.04 (0.02–0.14)	0.15	0.15	0.42 (0.23–0.69)	0.58	0.33 (0.22–0.42)	0.34
Periventricular zone	101.30 (74.79–161.59)	101.17	–	0.77 (0.61–0.83)	–	0.57 (0.25–1.68)	–

Abbreviations: CTV: clinical target volume; DLS: Deep learning segmentation; DSC; Dice similarity coefficient; GTV: gross tumor volume; HD95: 95th percentile Hausdorff distance. Volumes are given in cubic centimeters, whereas the unit for HD95 is centimeters.

**Table 3:** Median DSC, HD95, and volume size for all delineated structures for dummy run case 2. Target volumes and organs of interest are ranked from the largest to the smallest median volume. For all structures the consensus was used as reference.

Structure	Volume size (cm^3^) median (range)	Volume size (cm^3^) consensus	Volume size (cm^3^) DLS	DSC median (range)	DSC DLS	HD95 (cm) median (range)	HD95 (cm) DLS
CTV	348.02 (245.84–393.08)	377.17	–	0.92 (0.78–0.94)	–	0.65 (0.40–1.59)	–
GTV	88.99 (26.80–123.90)	106.03	–	0.86 (0.39–0.91)	–	0.52 (0.33–1.23)	–
Brainstem	29.69 (26.29–33.02)	30.28	27.38	0.92 (0.87–0.93)	0.90	0.24 (0.18–0.37)	0.24
Retina, right	2.34 (0.98–3.20)	3.65	–	0.64 (0.38–0.72)	–	0.24 (0.20–0.55)	–
Retina, left	2.36 (1.33–2.91)	3.78	–	0.63 (0.43–0.67)	–	0.25 (0.21–0.48)	–
Hippocampus, right	2.29 (0.49–3.27)	3.11	–	0.77 (0.12–0.83)	–	0.25 (0.21–1.05)	–
Hippocampus, left	2.47 (0.71–2.89)	2.92	–	0.77 (0.24–0.84)	–	0.23 (0.21–0.71)	–
Cornea, right	0.71 (0.45–1.14)	1.44	–	0.55 (0.31–0.71)	–	0.34 (0.18–0.72)	–
Cornea, left	0.73 (0.51–1.11)	1.35	–	0.54 (0.26–0.65)	–	0.29 (0.19–0.70)	–
Optic nerve, right	0.78 (0.50–1.04)	0.97	0.69	0.71 (0.64–0.83)	0.70	0.20 (0.17–0.24)	0.21
Optic nerve, left	0.70 (0.39–0.97)	0.99	0.66	0.70 (0.53–0.85)	0.72	0.20 (0.16–0.32)	0.22
Lacrimal gland, right	0.38 (0.10–1.28)	0.97	0.51	0.54 (0.19–0.78)	0.62	0.40 (0.24–0.97)	0.34
Lacrimal gland, left	0.34 (0.08–1.26)	0.65	0.51	0.64 (0.14–0.81)	0.78	0.70 (0.18–1.21)	0.16
Optic chiasm	0.42 (0.20–0.81)	0.60	0.30	0.64 (0.43–0.81)	0.55	0.28 (0.18–0.68)	0.44
Hypothalamus, right	0.44 (0.10–0.80)	0.30	–	0.39 (0.24–0.50)	–	0.59 (0.36–1.59)	–
Hypothalamus, left	0.42 (0.11–0.85)	0.29	–	0.45 (0.26–0.54)	–	0.60 (0.33–1.74)	–
Pituitary gland	0.28 (0.10–0.52)	0.41	0.48	0.69 (0.15–0.81)	0.69	0.31 (0.18–0.54)	0.29
Lens, right	0.18 (0.09–0.21)	0.21	0.15	0.78 (0.58–0.86)	0.78	0.14 (0.11–0.21)	0.12
Lens, left	0.18 (0.13–0.22)	0.22	0.15	0.78 (0.63–0.90)	0.78	0.14 (0.12–0.18)	0.14
Cochlea, right	0.06 (0.02–0.20)	0.19	0.17	0.47 (0.20–0.80)	0.60	0.28 (0.14–0.32)	0.32
Cochlea, left	0.07 (0.04–0.20)	0.18	0.20	0.55 (0.33–0.79)	0.46	0.19 (0.16–0.25)	0.47

Abbreviations: CTV: clinical target volume; DLS: Deep learning segmentation; DSC; Dice similarity coefficient; GTV: gross tumor volume; HD95: 95th percentile Hausdorff distance. Volumes are given in cubic centimeters, whereas the unit for HD95 is centimeters.

### 5.2 Target volumes

**Fig. 2:**
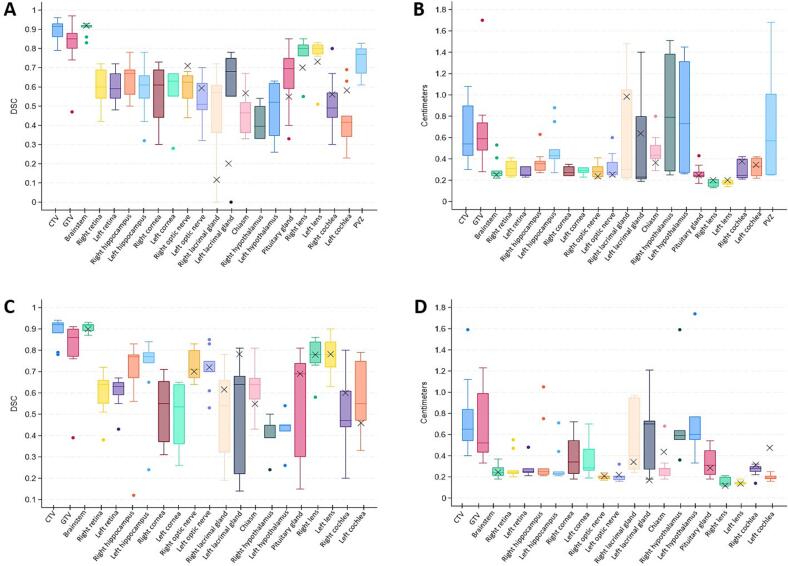
A displays dice similarity coefficient (DSC) for all participating study centers in the dummy run (DR) case 1, whereas C displays DSC for participating study centers in DR case 2. B displays the 95th percentile Hausdorff distance (HD95) for DR case 1, and D for DR case 2. Target volumes and organs of interest are ranked from the largest volume on the left side, to the smallest volume on the right side. The cross represents DSC for the deep learning segmentation. Horizontal lines of boxes represent the median and the first and third quartiles. Whiskers show the smallest and largest values within 1.5 times the interquartile range, whereas data points outside the whiskers are displayed as individual dots. CTV: clinical target volume; DSC: Dice similarity coefficient; GTV: gross tumor volume; HD95: 95th percentile Hausdorff distance; PVZ: periventricular zone.

**Fig. 3:**
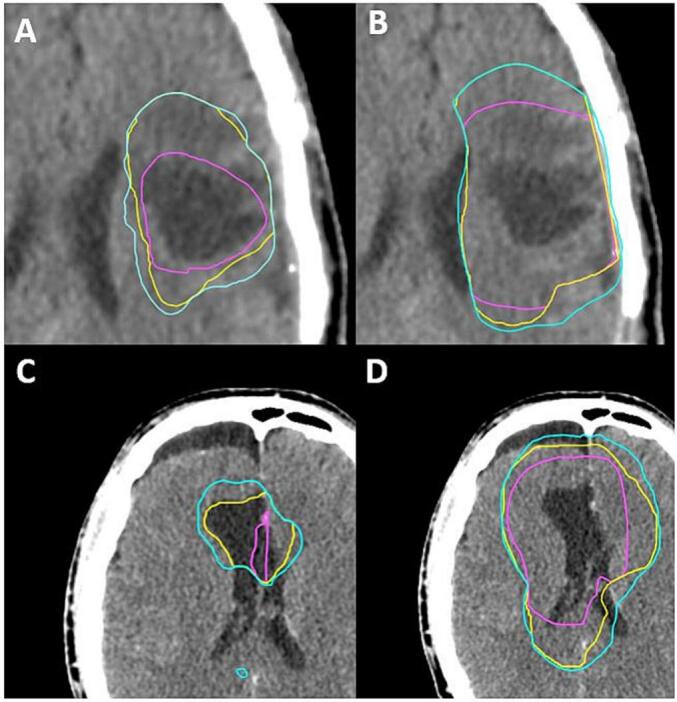
A shows gross tumor volume (GTV) and B clinical target volume (CTV) for dummy run (DR) case 1, while C displays GTV and D CTV for DR case 2. The pink line represents the minimal volume agreement among 10 delineators, the yellow line indicates the minimal volume agreement among 7 delineators, and the turquoise line shows the minimal volume agreement among 3 delineators. (For interpretation of the references to colour in this figure legend, the reader is referred to the web version of this article.)

Median DSC was 0.92 for clinical target volume (CTV) in both cases, whereas for gross tumor volume (GTV) median DSC was 0.85 (range 0.47–0.97) for case 1 and 0.86 (range 0.39–0.91) for case 2. Median HD95 was 0.59 cm and 0.54 cm for GTV and CTV in case 1, respectively, with corresponding values of 0.52 cm 0.65 cm in case 2. GTV size ranged between 23.99 and 75.30 cc for case 1, and 26.80–123.90 cc for case 2, whereas the CTV volume ranged between 143.23 and 221.99 cc for case 1 and 245.84–393.08 cc for case 2 (Table 2, Table 3, Fig. 1, Fig. 2, Fig. 3).

### 5.3 Organs of interest

The mandatory OOI with the highest DSC with a median of 0.92 for both cases was the brainstem, which was also the OOI with the largest delineated volume. For case 1 the left cochlea had the lowest DSC (median 0.42), followed by the chiasm (0.47). Median DSC was lowest for the right cochlea in case 2 (0.47), followed by the left cochlea (0.55). The PVZ in case 1 had a median DSC of 0.77 (range 0.61–0.83). HD95 was below 0.30 cm for all but one mandatory OOI in case 2, with the pituitary gland (0.31 cm) representing the only exception. For case 1 the left cochlea (0.33 cm) and the chiasm (0.44 cm) were the only mandatory OOI with HD95 above 0.30 cm.

For the optional OOI, the hypothalami had the lowest DSC in both cases (Table 2, Table 3, Fig. 2, Fig. 3). Median HD95 was 0.30 cm or lower for all optional OOI, excluding the hypothalami and the right retina in case 1 and the hypothalami, lacrimal glands, and the right cornea in case 2 (Table 2, Table 3, Fig. 2, Fig. 3).

### 5.4 Deep learning segmentation (DLS)

The DLS-based segmentations had a DSC that was equal to or closer to the consensus than the median DSC for manual delineations in six of 11 OOI and seven of 11 OOI in case 1 and 2, respectively. DSC for all DLS-based structures was within the ranges seen in manually delineated structures. For HD95 DLS had comparable or a value closer to the consensus than the median manual delineations in five OOI in case 1 and in six OOI in case 2 (Table 2, Table 3, Fig. 1, Fig. 2, Fig. 3).

### 5.5 Dummy run questionnaire

The applied margin from GTV to CTV in case 1 was 10 mm for seven study centers (as suggested by the PRO-GLIO protocol for grade 2 tumors), 12 mm for two centers, and 15 mm for one center. For the second case, all delineators applied a margin of 15 mm from GTV to generate CTV as is also suggested in the PRO-GLIO protocol for grade 3 tumors. All delineators adjusted target volumes for anatomical barriers.

### 5.6 Treatment plan evaluation

The homogeneity index for CTVs was median 0.03–0.05 (optimal value 0) for all four plans, whereas the Radiation Therapy Oncology Group conformity index for CTVs was median 1.27–1.39, which was deemed acceptable (see Supplementary Table 5 for remaining values). Volumetric modulated arc therapy (VMAT) was used in all photon treatment plans in both cases. For proton plans, five centers applied multi-field optimization (MFO) and four centers single-field optimization (SFO) in case 1, whereas for case 2 five centers applied MFO and two centers SFO.

**Supplementary Fig. S3:**
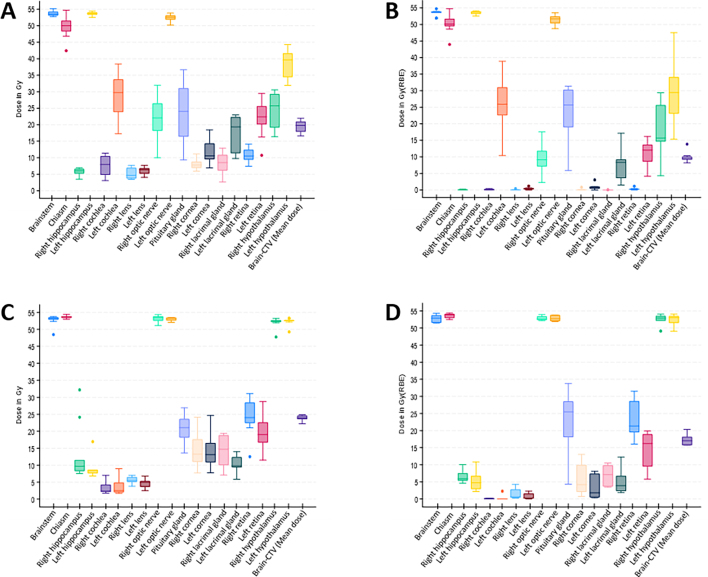
Boxplots showing doses delivered to organs of interest assessing interobserver variations in treatment planning across different study centers.

Dose constraints to OOI were fulfilled in both cases, with some exceptions: the pituitary gland received ≥20 Gy relative biological effect (RBE) in two thirds of the proton and photon plans in case 1, and five of nine photon plans and five of seven proton plans in case 2. Due to the anatomical location of the target volume in case 1, high doses were delivered to the left hippocampus in all treatment plans. For case 2, none of the photon plans managed to fulfill dose constraints to the right hippocampus (<7.3 Gy(RBE) to D_40%_) and only one plan was below the dose constraint to the left hippocampus. For the corresponding proton plans in case 2, the right hippocampus received ≥7.3 Gy(RBE) to D_40%_ in two plans and for the left hippocampus in one plan. In addition, all photon and proton plans in case 2 had mean doses to the hypothalami >45 Gy(RBE) (Supplementary Table 6 and Supplementary Fig. 3).

**Supplementary Fig. S4:**
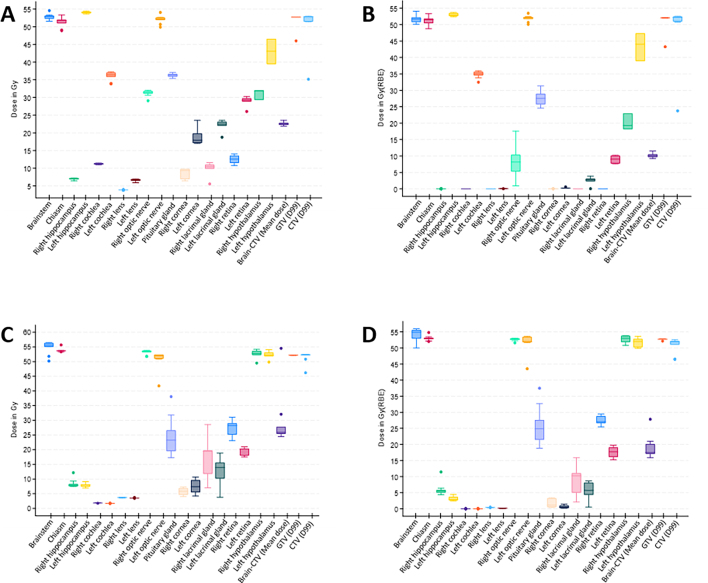
Differences in dose delivered to organs of interest (OOI) and target volumes using the same treatment plan applied to all study centers´ delineations.

When evaluating the dosimetric impact of applying the same treatment plan across different centers´ delineations, dose variations to most OOI were small and remained within established dose constraints (Supplementary Table 7). For serial OOI (brainstem and optic apparatus), dose constraints were fulfilled in all four plans, except the optic chiasm for one center in the photon plan for case 1. Mean dose to brain minus CTV (Brain-CTV) differed by as much as 6.2 and 12.0 Gy(RBE) in VMAT and PBT plans for case 2, respectively. In contrast, smaller differences were seen for Brain-CTV in case 1, with maximum variations of 1.7 and 2.3 Gy(RBE), respectively. For CTV, there was some variation. In three treatment plans, only one of the delineated CTVs received less than the prescribed dose (51.3 Gy(RBE) to 99% of CTV (D_99%_)), whereas four of 10 delineated CTVs had suboptimal coverage in the fourth plan. For two CTVs D_99%_ was 51.0 and 51.2 Gy(RBE) which was just below the prescribed dose (>51.3 Gy(RBE) to D_99%_), for the two other CTVs in the same plan the dose was 46.5 and 46.6 Gy(RBE) to D_99%_, and in three remaining cases the underdosed CTV had a D_99%_ of 46.2 Gy, 35.2 Gy, and 23.8 Gy(RBE) (Supplementary Table 7 and Supplementary Fig. 4).

## 6 Discussion

In this DR procedure, contouring consistency was especially good for CTV with a median DSC of 0.92 for both cases. Although there was some variation for HD95, this is reassuring as CTV is the main target volume in *IDH*-mutated gliomas. Volume, median DSC and median HD95 values varied for most OOI, and for some of them considerably. Whereas variation in delineation of non-standard OOI is understandable, the substantial variation for routinely delineated OOI such as the chiasm is of more concern. DLS-based segmentation was within the range of manual delineations, illustrating the future potential of DLS. Radiotherapy plans were based on consensus delineations to exclude the impact of delineation discrepancies, and there was only minor variability.

Results from interventional clinical studies depend highly on the quality of all involved procedures, this includes possible interobserver variations and studies on effect and side effects of radiotherapy are no exception [4], [29], [30]. Factors such as the experience of delineators and available imaging modalities might influence interobserver variations [5], [31], [32]. Such variations are known from other studies, such as the DR procedure for the EORTC low-grade glioma trial 22,033–26,033, where two centers had to repeat the target volume delineations and some OOI were missing in one third of the plans [33]. In the same trial, later individual case reviews for 57 cases showed that 25% of mandatory OOI were missing, up to one-third of present OOI were incorrectly delineated, and just over 30% had satisfactory target volume definitions [34]. In our study, the most experienced radiation (neuro)-oncologists across twelve institutions in two Scandinavian countries participated. All had the same patient and imaging information available. We found a higher DSC variability for small OOI compared to large OOI, as has also been shown by others [35], [36], [37]. For example, our findings of a DSC of 0.92 for the brainstem in both cases, and 0.47 and 0.64 for the chiasm, correlates well with Deeley et al. and Lorenzen et al. [36], [37]. Following reports on higher incidence of radiation induced contrast enhancement (RICE) in the PVZ in *IDH*-mutated gliomas treated with proton therapy compared to photon radiotherapy [38], [39], although others studies have reported a more comparable incidence [40], [41], [42], [43], [44], the PVZ was added as a mandatory OOI at a later stage. We found a DSC of 0.77 for the PVZ, however, slice-by-slice evaluation demonstrated larger discrepancies and a workshop within the PRO-GLIO trial followed to improve consistency.

Delineation of target volumes for diffuse gliomas in general is complex, not least considering the microscopic tumor infiltration, making contouring dependent on subjective considerations [45]. Our observation of a better DSC for CTV than for GTV is probably related to the DSC definition favoring larger volumes, and the use of HD95 balances this and revealed some variation. Nonetheless, the generally good agreement on CTV delineation between experts was reassuring. This is comparable to a large multi-institutional dummy run procedure conducted by Ono et al., where 41 institutions delineated GTV and CTV on a glioblastoma case. Mean DSC was 0.37 for GTV and 0.80 for CTV in that study [46].

We found that the size of delineated structures varied considerably. GTV and CTV varied by a factor of 3.1–4.6 and 1.5–1.6, respectively. This variation was slightly larger than data published by Weltens et al. [47], but smaller than what was found by Boer et al. before the latter organized a consensus meeting [48]. For OOI, volume variations in our study were larger than for target volumes. Such variations may clearly impact treatment results, including toxicity. Interestingly, practically all consensus delineations were larger than corresponding median volumes from the study centers. Discrepancies in volume size across the expert group were also seen in the independent delineations prior to the consensus meeting (Supplementary Table 4), underscoring that the consensus delineation did not represent the ground truth but merely a reference.

Interestingly, structures generated by DLS were within the range of manual delineations, similar to results reported by others [13], [14], [15], [36], [49]. The potential DLS might bring about is very appealing, saving resources and introducing consistency. However, with the relatively large variation in manual delineations, merely being within the human range does not render DLS outstanding. It also needs to be emphasized that only one DLS model was applied in this study and for a limited number of OOI, and DLS was not available for target volumes. Several studies are investigating the potential of DLS-based target volume delineations, however, further validation for several OOI and target volume DLS definition is needed before implementation in clinical practice [13], [17], [50].

Satisfactory conformity and heterogeneity indices for all radiotherapy plans were seen in our study, with only minor variation in doses to OOI. This is reassuring and leads us to believe that target and OOI delineation is the area with the highest potential for improvement. The decision to use consensus delineations to exclude interobserver variability for the planning study is a strength for evaluation of planning variation, however, also a limitation as the impact of delineation variability on radiotherapy plans could not be assessed. However, to evaluate the possible clinical impact of the interobserver variation in delineation, all study centers delineations were imported into the same treatment plan. Although some variation was seen, most OOI were well within dose constraints. More concerning was the underdosage to CTV seen in seven scenarios, of which four were in the PBT plan for case 2.

All study centers were approved for patient inclusion following the DR procedure. Although a debatable conclusion based on the observed delineation variability, this reflects acceptance of the real-world situation. We believe this DR procedure underscores the critical role of QA in radiotherapy trials, facilitating increased focus and discussion on precise delineation. Ideally, the dummy run procedure could have been repeated on the same two cases following feedback to and subsequent discussions between the delineators.

This study has limitations. DSC has limitations based on the volume of analysed structures, however, this was balanced using HD95. Only two cases were included and only one was mandatory. Furthermore, the consensus delineation is not a ground truth, rather a basis for comparison. The number of delineators was also not very high; however, the delineators were experts representing all 12 relevant treatment facilities in Norway and Sweden which is a major strength.

**The following are the supplementary data related to this article.**

Supplementary material 5 Supplementary figure legends IMAGE 5

Supplementary material 6 PRO-GLIO study; Manual for the dummy run procedure. IMAGE 6

Supplementary material 7 Participating centers in the dummy run procedure. IMAGE 7

Supplementary material 8 Organs of interest to be delineated. IMAGE 8

Supplementary material 9 Parameters used assessing target volumes. IMAGE 9

Supplementary material 10 Volume size of expert structures for dummy run case 1 and 2, including inter-expert range. IMAGE 10

Supplementary material 11 Treatment plan evaluation of target volumes with median values (range) IMAGE 11

Supplementary material 12 Median dose (range) to organs of interest IMAGE 12

Supplementary material 13 Differences in dose delivered to organs of interest (OOI) and target volumes were evaluated using the same treatment plan applied to all study centers’ delineations. Median dose (range) across study centers for OOI is reported, based on VMAT and PBT treatment plans developed at Oslo University Hospital for both cases. IMAGE 13

## Ethics

The PRO-GLIO trial has been approved by ethical committees in Norway (Regional Committee for Medical & Health Research Ethics, South East Norway, Section C: reference number: 265626) and Sweden (The Swedish Ethical Review Authority, Västra Götaland: reference number: Dnr 2021–04239 and Dnr 2022–01305-02) before trial start. The trial will adhere to the ethical recommendations of the International Committee of Harmonisation-Good Clinical Practice guidelines, the Helsinki declaration and Norwegian and Swedish laws and regulations.

## Funding

10.13039/501100006095South-Eastern Norway Regional Health Authority (Project number: 2021081); The 10.13039/100008730Norwegian Cancer Society (Project number: 216158); Network in Radiation Oncology (NIRO); The 10.13039/501100007687Swedish Society of Medicine (SLS-890541); The Gothenburg Society of Medicine (GLS-887961); Jubileumsklinikens Cancerfond; Lions Cancer Research Fund of Western Sweden.

## Declaration of competing interest

The authors declare that they have no known competing financial interests or personal relationships that could have appeared to influence the work reported in this paper.
