# Supplementary material for: Radiotherapy quality assurance in the PRO-GLIO trial: results from a dummy run comparing experts across twelve institutions in two Scandinavian countries
Source: Clin Transl Radiat Oncol. 2026 Jun 18;60:101220. doi: 10.1016/j.ctro.2026.101220 (PMC13316294; doi:10.1016/j.ctro.2026.101220)
Supplement: Supplementary material 5 — Supplementary figure legends [file mmc5.docx]

**Supplementary Figures**

Supplementary Figure 1: Figure A displays axial dose planning CT images for case 1 and Figure B from case 2. Consensus delineations are depicted with gross tumor volume in orange, clinical target volume in red, brainstem in brown, optic nerves in green, chiasm in blue, hippocampi in green, hypothalami in pink, lenses in blue, corneae in yellow, retinae in orange, and lacrimal glands in brown.

Supplementary Figure 2: In A the dice similarity coefficient (DSC) quantifying volumetric overlap between volumes, calculated using the formula: $DSC=\frac{2\left| A\cap B \right|}{\left| A \right|+\left| B \right|}$, is visualized. In B the 95th percentile Hausdorff distance (HD95) is visualized. HD95 is a robust surface distance metric that represents the 95th percentile of the shortest distances between points on one contour and their corresponding points on the compared contour. HD95 provides a reliable measure of spatial deviation by excluding the most extreme 5% of point-to-surface distances, thereby minimizing the influence of outliers.

Supplementary Figure 3: Boxplots showing doses delivered to organs of interest assessing interobserver variations in treatment planning across different study centers. All centers used consensus delineations for their treatment planning. Figure A and B show results from photon and proton plans for dummy run case 1, respectively, and C and D similarly for case 2. For hippocampi the depicted dose corresponds to the minimum dose delivered to 40% of the delineated volume. For cochleae, pituitary gland, lacrimal glands, and hypothalami mean doses are shown, and for the remaining organs of interest dose to 0.03 cubic centimeters (maximum dose) is given.

CTV: clinical target volume; GTV: gross tumor volume; Gy: Gray; RBE: relative biological effect; V_30Gy_: volume receiving 30 Gy.

Supplementary Figure 4: Differences in dose delivered to organs of interest (OOI) and target volumes were evaluated using the same treatment plan applied to all study centers´ delineations. Figure A and B show doses for the photon and proton plan in dummy run case 1, respectively, and C and D the corresponding doses for the photon and proton plan in case 2. For the hippocampi depicted doses corresponds to the minimum dose delivered to 40% of the delineated volume. Mean doses are reported for cochleae, pituitary gland, lacrimal glands, and hypothalami,and for the remaining OOI dose to 0.03 cubic centimeters (maximum dose) is shown. For all volumes doses in Gy/Gy(RBE) are depicted.

CTV: clinical target volume; D_99%_: %: the minimum dose received by 99% of the volume; GTV: gross tumor volume; Gy: Gray; RBE: relative biological effect; V_30Gy_: volume receiving 30 Gy.

**Supplementary Tables**

Supplementary Table 1. Participating centers in the dummy run procedure

Supplementary Table 2. List of mandatory and optional organs of interest to be delineated

Supplementary Table 3: Parameters used assessing target volumes

Supplementary Table 4. Volume size of expert structures for dummy run case 1 and 2, including inter-expert range

Supplementary Table 5: Treatment plan evaluation of target volumes with median values (range)

Supplementary Table 6: Treatment plan evaluation of organs of interest with median dose (range)

Supplementary Table 7: Differences in dose delivered to organs of interest (OOI) and target volumes were evaluated using the same treatment plan applied to all study centers’ delineations. Median dose (range) across study centers for OOI is reported, based on VMAT and PBT treatment plans developed at Oslo University Hospital for both cases.

**Supplementary material**

Supplementary Material 1: Dummy run manual including dummy run questionnaire
