# Supplementary material for: Radiotherapy quality assurance in the PRO-GLIO trial: results from a dummy run comparing experts across twelve institutions in two Scandinavian countries
Source: Clin Transl Radiat Oncol. 2026 Jun 18;60:101220. doi: 10.1016/j.ctro.2026.101220 (PMC13316294; doi:10.1016/j.ctro.2026.101220)
Supplement: Supplementary material 6 — PRO-GLIO study; Manual for the dummy run procedure. [file mmc6.pdf]

PRO-GLIO-study

Manual for the dummy run procedure

Procedure for the implementation and approval of delineation of target volumes and organs at risk and development of dose plans prior to the start of a study center in the PRO-GLIO study.

1. Background

The purpose of conducting the dummy run (DR) procedure is quality assurance of the radiotherapy deliverance in the PRO-GLIO study across participating centers. The PRO-GLIO DR consists of two cases. Structures must be delineated according to the protocol, and a dose plan must be developed. To enable comparison of dose plans between centers, the plans must be based on the same structure set (see section 4).

2. Execution of the Dummy Run

The dummy run will be conducted as a two-step process:

1. **Step 1** concerns the delineation of target volumes and organs at risk. First, the specified structures are to be delineated by following the delineation guidelines (see below). It is not necessary to add the PTV, but the margin typically used from CTV to PTV must be specified. It is desirable that the delineating oncologist answers the three questions in Appendix 1 of this document.
2. **Step 2** concerns planning. Based on the consensus case with pre-delineated target volumes and organs at risk, all centers must develop a photon plan in accordance with the protocol. Swedish centers must also develop a proton plan, for the Norwegian centers not having a proton facility at their hospital this is optional.

3. DICOM Data

Each case includes CT, preoperative T1 post-contrast MRI series, preoperative T2/FLAIR MRI series, postoperative T1 post-contrast MRI series, postoperative T2/FLAIR MRI series, and a structure set with standardized naming of ROIs according to the PRO-GLIO protocol. A consensus-based structure set is also available and must be used for radiotherapy planning. Additionally, an Excel file is included with standard names for all PRVs not included in the structure set.

Note (applies to Step 1): To ensure naming of ROIs according to the protocol, a structure set has been exported from the dose planning system to ShareFile, where each ROI has a volume. In the attached structure set for each dummy run case, there is a delineation in one slice for all ROIs. This delineation must be removed before starting your own delineation.

4. Case Descriptions

In the folder for the DR procedure you will find documents describing each individual case. Here you also have access to all cases, including a consensus case with pre-delineated target volumes and organs at risk.

5. Delineation Guidelines

1. **OAR_delineation_guidelines_Technical_document16_rev.pdf** is a document with the current Norwegian delineation guidelines. The development of these national guidelines is organized by the KVIST group (Quality Assurance in Radiotherapy) at the Norwegian Radiation and Nuclear Safety Authority.

2. **OAR_inntegningsveiledning_v2.pdf.** This is an appendix to the technical document containing detailed information about the recommendations.

6. Uploading of Delineation and Dose Plan

Upon completion of delineation and planning, the structure set (**NB! Including CT**), plan, and plan dose (not beam dose) in DICOM format must be uploaded.

After the upload is complete, a notification should be sent to the study team at Oslo University Hospital.

The person(s) receiving the upload notification for the delineation and/or radiotherapy plans will forward the information to the remaining members of the PRO-GLIO team. And at least on oncologist in the responsible team must review **Appendix 2** before the dummy run can be approved.

Petter Brandal will notify the principal investigator at each center of the results as soon as the evaluation is complete.

7. Approval of “Dummy Run” Dose Plans

Center Name:

The following dose plans have been submitted for evaluation:

Case	Delineation Submission date	Approved Initials and Date of Evaluation	Photon Plan Submission Date	Approved Initials and Date of Evaluation	Proton Plan Submission Date	Approved Initials and Date of Evaluation
DR 1		YES / NO		YES / NO		YES / NO
DR 2		YES / NO		YES / NO		YES / NO

Final Evaluation:

Appendix 1

Oncologist Questions – Dummy Run Patients

1. **What margin did you choose from GTV to CTV?**
   ☐ 10 mm ☐ 15 mm ☐ 20 mm
   a. *What is the rationale for choosing this specific margin?*

2. **Did you make any adjustments to the automatically generated CTV (enlarged or reduced in any direction)?**
   ☐ Yes ☐ No
   a. *Why did you make this change?*

3. **Do you think this patient was a good candidate for PRO-GLIO?**
   ☐ Yes ☐ No ☐ Don’t know
   a. *What is the rationale for your answer?*

4. **Are the doses to the OARs EQD2-adjusted?**
   ☐ Yes ☐ No

Appendix 2

Checklist:

I. Delineation

a. Target Volumes

- ☐ All delineated
- ☐ Acceptable delineation

Comments:

b. Organs at Risk (OARs)

- ☐ All delineated
- ☐ Acceptable delineation

Comments:

II. Radiotherapy Plan

a. Photon Plan

- ☐ Acceptable plan

b. Proton Plan

- ☐ Acceptable plan

Comments:

Criteria for Target Volume Delineation:

1. **Dice Similarity Coefficient (DSC):**
    - DSC for **GTV** should preferably be above **0.7**
    - DSC for **CTV** should preferably be above **0.8**, compared to the corresponding consensus volumes
2. **Correct margin** (as per protocol) from **GTV** to generate **CTV**
3. **Trimming against natural anatomical barriers:**
   a. Midline/Falx
   b. Tentorium
   c. Skull

DSC:

- GTV: ______
- CTV: ______

Criteria for Organ-at-Risk (OAR) Delineation:

1. The following **MUST** be delineated:
   a. Brainstem (including **surface** and **core/interior**)
   b. Cochleae
   c. Eyes
   d. Hippocampi
   e. Lenses
   f. Chiasm
   g. Optic nerves
   h. Pituitary gland
   i. Skin
2. The following are **optional**:
    - Corneas
    - Hypothalamus
    - Lacrimal glands
    - Spinal cord
    - Retinae
3. **Special attention should be given to the following OARs** (qualitatively most important, but DSC should also be considered):
   a. Brainstem surface – preferably **above 0.8**
   b. Chiasm – preferably **above 0.5**
   c. Hippocampus – preferably **above 0.5**

DSC:

- Brainstem Surface: ______
- Chiasm: ______
- Hippocampus_R: ______
- Hippocampus_L: ______

Criteria for the Treatment Plan:

1. **Dose Coverage of Target Volume**
   a. Assess whether the criteria in the protocol are fulfilled
   a. **Photons:** The 95% isodose should cover the PTV, alternatively **D98 > 95%**
   b. **Protons:** The 95% isodose **must** cover the CTV for all robustness evaluations (**±3 mm and ±3% range**)
   c. Underdosing is acceptable **if necessary to prioritize OARs**
2. • **Field Setup**
   a. Semi-qualitative assessment
3. • **Dose Constraints for Organs at Risk (OARs)**
   a. Check whether the criteria specified in the table below (**Table 3 in the protocol**) are met
   (Conversion to EQD2 only if this is the institution's standard practice)
4. • **Verify that PRV margins** correspond to the institution's standard practice
5. • **For Proton Plans:** Check that the **geometric and range uncertainties** used in robust optimization correspond to the **margins applied when expanding from CTV to PTV and from OAR to PRV**.

Dose Constraints Table (EQD2, RBE-adjusted)

Priority	Organ	α/β	Dose Constraint (EQD2, RBE)	Toxicity	Criterium Fulfilled or Not
1	Chiasm & Optic Nerves	2	$D_{0.03cc}$ ≤ 55 Gy	Optic neuropathy	
2	PRV Chiasm & Optic Nerves		$D_{2\%}$ ≤ 60 Gy		
3	Brainstem Surface	2	$D_{0.03cc}$ ≤ 60 Gy	Permanent cranial neuropathy or necrosis	
	Brainstem Interior	2	$D_{0.03cc}$ ≤ 54 Gy	Permanent cranial neuropathy or necrosis	
4	Spinal Cord		$D_{2\%}$ ≤ 50 Gy	Palsy	
5	PRV Spinal Cord		$D_{2\%}$ ≤ 54 Gy		
6	GTV		$D_{2\%}$ ≤ 107% of prescribed dose		
7	CTV		$D_{2\%}$ ≤ 107% of prescribed dose		
8	PTV (Photons) / Robust CTV (Protons)		$D_{98\%}$ ≥ 95% of prescribed dose $D_{2\%}$ ≤ 107% of prescribed dose		
9	Retina	3	$D_{0.03cc}$ ≤ 45 Gy	Loss of vision	
10	Cochlea	3	D_{mean} ≤ 45 Gy	Hearing loss	
11	Pituitary	2	D_{mean} ≤ 45 Gy	Panhypopituitarism	
	Pituitary	2	D_{mean} ≤ 20 Gy	Growth hormone deficiency	
12	Lens	1	$D_{0.03cc}$ ≤ 10 Gy	Cataract	
13	Lacrimal Gland	3	D_{mean} ≤ 25 Gy	Keratoconjunctivitis sicca	
14	Hippocampus	2			
