# Supplementary material for: Radiotherapy quality assurance in the PRO-GLIO trial: results from a dummy run comparing experts across twelve institutions in two Scandinavian countries
Source: Clin Transl Radiat Oncol. 2026 Jun 18;60:101220. doi: 10.1016/j.ctro.2026.101220 (PMC13316294; doi:10.1016/j.ctro.2026.101220)
Supplement: Supplementary material 7 — Participating centers in the dummy run procedure. [file mmc7.docx]

Supplementary Table 1. Participating centers in the dummy run procedure

| **Trial Centre** | **Location** |
| --- | --- |
| Department of Oncology, Oslo University Hospital | Oslo, Norway |
| Department of Oncology, Sahlgrenska University Hospital | Gothenburg, Sweden |
| Department of Oncology, University Hospital of North Norway | Tromsø, Norway |
| Department of Oncology, St. Olavs University Hospital | Trondheim, Norway |
| Department of Oncology, Haukeland University Hospital | Bergen, Norway |
| Department of Oncology, Lund University Hospital | Lund, Sweden |
| Department of Oncology, Örebro University Hospital | Örebro, Sweden |
| Department of Oncology, Umeå University Hospital | Umeå, Sweden |
| Department of Oncology, Uppsala University Hospital | Uppsala, Sweden |
| Department of Oncology, Karolinska University Hospital | Stockholm, Sweden |
| Department of Oncology, Linköping University Hospital | Linköping, Sweden |
| Department of Oncology, Jönköping University Hospital | Jönköping, Sweden |
