# Supplementary material for: Radiotherapy quality assurance in the PRO-GLIO trial: results from a dummy run comparing experts across twelve institutions in two Scandinavian countries
Source: Clin Transl Radiat Oncol. 2026 Jun 18;60:101220. doi: 10.1016/j.ctro.2026.101220 (PMC13316294; doi:10.1016/j.ctro.2026.101220)
Supplement: Supplementary material 8 — Organs of interest to be delineated. [file mmc8.docx]

Supplementary Table 2. Organs of interest to be delineated

| **Mandatory** | **Optional** |
| --- | --- |
| Brain | Corneae |
| Brain stem (including surface and core/interior) | Hypothalami |
| Cochleae | Lacrimal glands |
| Eyes | Spinal cord |
| Hippocampi | Retinae |
| Lenses |  |
| Optic chiasm |  |
| Optic nerves |  |
| Periventricular zone (at a later stage) |  |
