# Supplementary material for: Radiotherapy quality assurance in the PRO-GLIO trial: results from a dummy run comparing experts across twelve institutions in two Scandinavian countries
Source: Clin Transl Radiat Oncol. 2026 Jun 18;60:101220. doi: 10.1016/j.ctro.2026.101220 (PMC13316294; doi:10.1016/j.ctro.2026.101220)
Supplement: Supplementary material 9 — Parameters used assessing target volumes. [file mmc9.docx]

Supplementary Table 3: Parameters used assessing target volumes

| Parameter | Formula* | Optimal value | Interpretation | Illustration |
| --- | --- | --- | --- | --- |
| Homogeneity index ^1^ | $\frac{D2\%-D98\%}{D50\%}$ | 0 | A homogeneity index of zero indicates that the absorbed dose distribution is homogenous. | 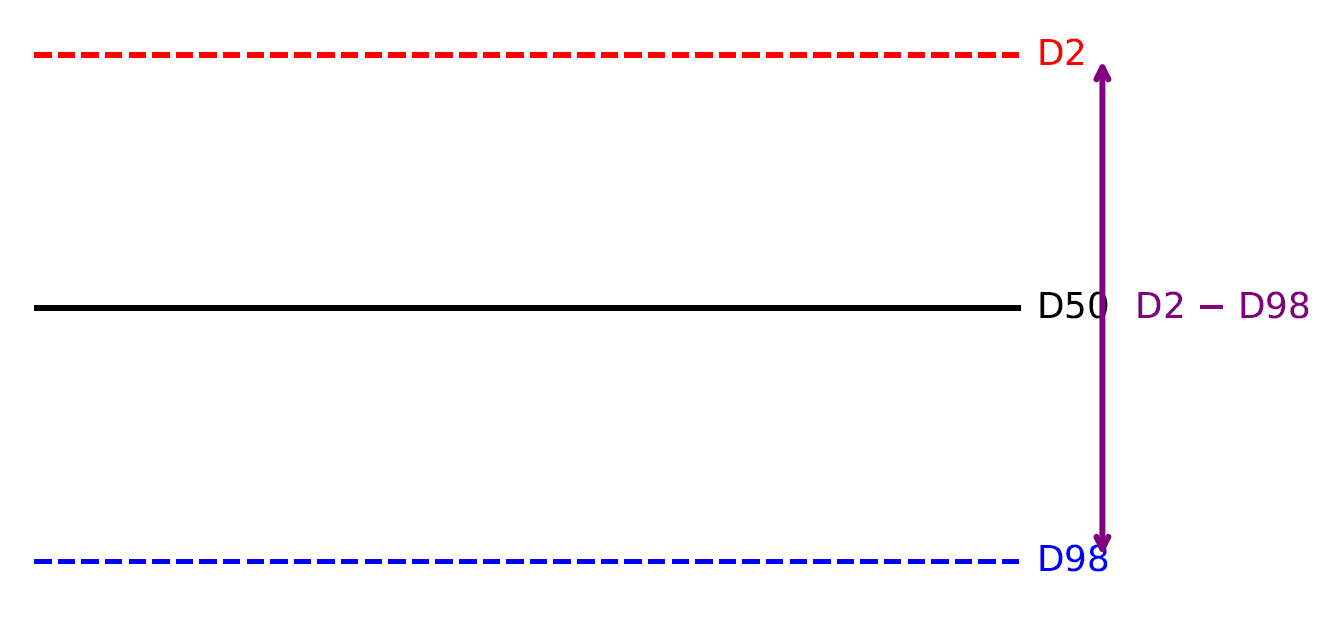 |
| RTOG conformity index ^2^ | $\frac{\mathrm{PIV}}{\mathrm{PTV}}$  &  $\frac{\mathrm{PIV}}{\mathrm{CTV}}$ | 1 | Measures how much of the prescription dose (V95%) covers the target volume. A value of 1 corresponds to ideal conformity. | 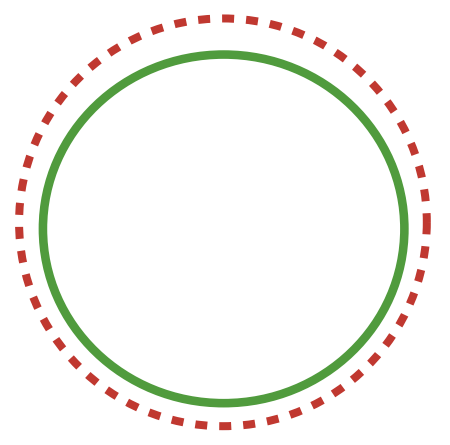 |
| Paddick/Van’t Riet conformity number ^3,4^ | $\frac{{PTV95}^{2}}{\mathrm{PTVxPIV}}$  &  $\frac{{CTV95}^{2}}{\mathrm{CTVxPIV}}$ | 1 | Measures the overlap between the target volume and prescription dose volume relative to the total irradiated volume. Dose spills and under-coverage are considered. | 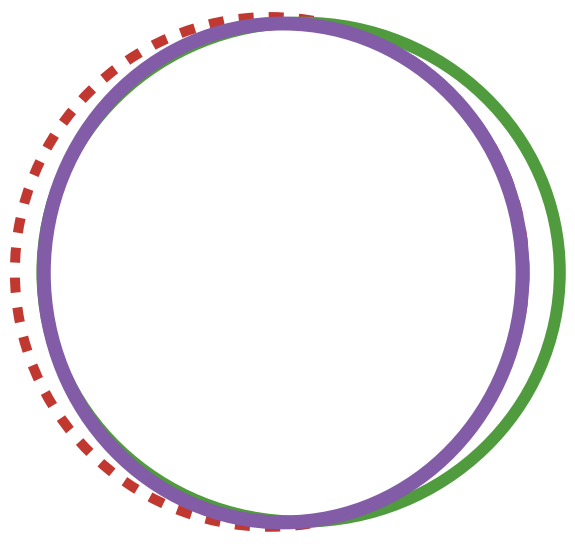 |
| Lesion coverage volume factor ^2^ | $\frac{PTV95}{\mathrm{PTV}}$  &  $\frac{CTV95}{\mathrm{CTV}}$ | 1 | The proportion of the target volume that receives at least the prescribed dose. | 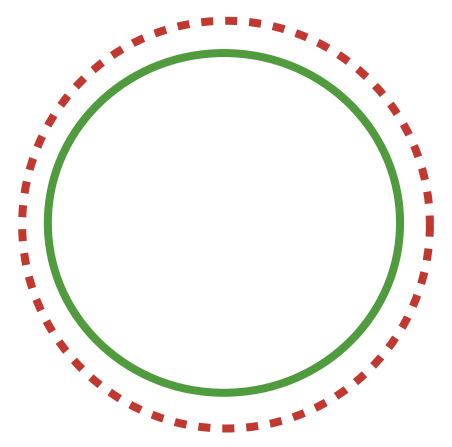 |
| Healthy tissues conformity index ^2,5^ | $\frac{PTV95}{\mathrm{PIV}}$  &  $\frac{CTV95}{\mathrm{PIV}}$ | 1 | To what degree healthy brain tissue outside the target volume is included in the reference isodose. | 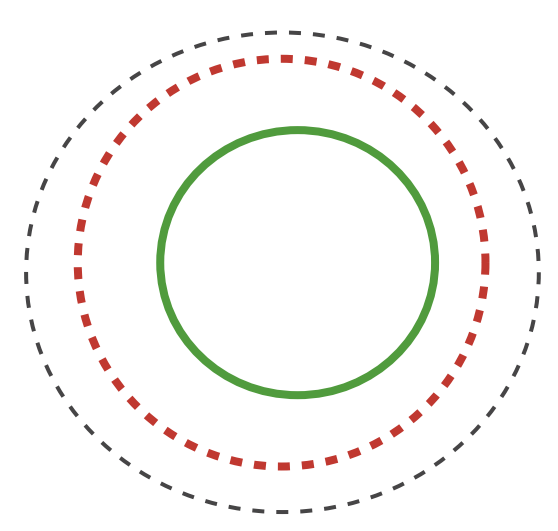 |
| CTV: clinical target volume; CTV95: clinical target volume receiving ≥95% of the total dose; D2: minimum dose to 2% volume of target volume; D50: median dose to target volume; D98: minimum dose to 98% volume of target volume; PIV: prescribed dose volume (defined as volume receiving 95% of the isodose); PTV: planning target volume; PTV95: planning target volume receiving ≥95% of the total dose; RTOG: Radiation Therapy Oncology Group  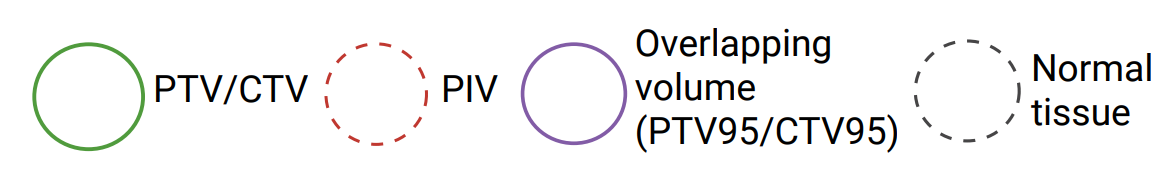  * For photon plans the isodose should cover the PTV, whereas for protons robust optimizing using 3 +/- 3 mm, +/- 3.5% range for most centers was applied, PTV with a 3 mm margin from the CTV were therefore used as a surrogate when assessing tumor volume coverage and conformity. However, this may have several faults, and we therefore also assessed the same parameters for the CTV, which in turn might provide inaccuracies as it always should be covered by the isodose to a higher degree than the PTV. To be able to compare proton versus photon we assessed all parameters for both PTV and CTV for both photon and proton plans. | | | | |

1. ICRU. ICRU, ed. *ICRU Vol. 10. No 1. Report 83. The International Commission on Radiation Units and*

*Measurements. Prescribing, recording, and reporting photon-beam IMRT.* 2010.

2. Feuvret L, Noël G, Mazeron J-J, Bey P. Conformity index: A review. *International Journal of Radiation Oncology*Biology*Physics*. 2006/02/01/ 2006;64(2):333–342. doi:<https://doi.org/10.1016/j.ijrobp.2005.09.028>

3. Riet Avt, Mak ACA, Moerland MA, Elders LH, van der Zee W. A conformation number to quantify the degree of conformality in brachytherapy and external beam irradiation: Application to the prostate. *International Journal of Radiation Oncology*Biology*Physics*. 1997/02/01/ 1997;37(3):731–736. doi:<https://doi.org/10.1016/S0360-3016(96)00601-3>

4. Paddick I. A simple scoring ratio to index the conformity of radiosurgical treatment plans. Technical note. *J Neurosurg*. Dec 2000;93 Suppl 3:219–22. doi:10.3171/jns.2000.93.supplement

5. Lomax NJ, Scheib SG. Quantifying the degree of conformity in radiosurgery treatment planning. *International Journal of Radiation Oncology, Biology, Physics*. 2003;55(5):1409–1419. doi:10.1016/S0360-3016(02)04599-6
