# Supplementary material for: Radiotherapy quality assurance in the PRO-GLIO trial: results from a dummy run comparing experts across twelve institutions in two Scandinavian countries
Source: Clin Transl Radiat Oncol. 2026 Jun 18;60:101220. doi: 10.1016/j.ctro.2026.101220 (PMC13316294; doi:10.1016/j.ctro.2026.101220)
Supplement: Supplementary material 10 — Volume size of expert structures for dummy run case 1 and 2, including inter-expert range. [file mmc10.docx]

Supplementary Table 4: Volume size of expert structures for dummy run case 1 and 2, including inter-expert range

| **Structure** | **Dummy run case 1 Consensus volume (range)** | **Dummy run case 2**  **Consensus volume (range)** |
| --- | --- | --- |
| CTV | 212.20 (194.12-296.65) | 377.17 (344.84-383.46) |
| GTV | 76.54 (45.86-90.50) | 106.03 (77.03-119.19) |
| Brainstem | 27.65 (26.08-28.60) | 30.28 (25.08-33.52) |
| Retina, right | 3.77 (2.94-4.06) | 3.65 (2.51-3.89) |
| Retina, left | 3.37 (2.81-3.40) | 3.78 (2.37-3.31) |
| Hippocampus, right | 1.83 (1.62-2.26) | 3.11 (0.97-3.00) |
| Hippocampus, left | 1.79 (1.55-2.33) | 2.92 (0.96-3.29) |
| Cornea, right | 1.16 (1.10-3.63) | 1.44 (0.53-2.00) |
| Cornea, left | 1.14 (1.08-2.22) | 1.35 (0.48-2.12) |
| Optic nerve, right | 0.58 (0.35-0.78) | 0.97 (0.77-0.97) |
| Optic nerve, left | 0.64 (0.30-0.90) | 0.99 (0.78-1.08) |
| Lacrimal gland, right | 0.36 (0.22-0.61) | 0.97 (0.37-1.08) |
| Lacrimal gland, left | 0.48 (0.35-0.78) | 0.65 (0.42-0.88) |
| Optic chiasm | 0.49 (0.44-1.15) | 0.60 (0.28-0.66) |
| Hypothalamus, right | 0.52* | 0.30 (0.11-0.32) |
| Hypothalamus, left | 0.49* | 0.29 (0.10-0.31) |
| Pituitary gland | 0.28 (0,.21-0.42) | 0.41 (0.16-0.66) |
| Lens, right | 0.26 (0.22-0.43) | 0.21 (0.19-0.30) |
| Lens, left | 0.25 (0.19-0.38) | 0.22 (0.19-0.32) |
| Cochlea, right | 0.13 (0.09-0.22) | 0.19 (0.10-0.22) |
| Cochlea, left | 0.15 (0.08-0.25) | 0.18 (0.10-0.20) |
| Periventricular zone | 101.17 (99.95-105.10) | Not applicable |

Abbreviations: CTV: clinical target volume; GTV: gross tumor volume. Volumes are given in cubic centimeters. *Range is missing.
