# Supplementary material for: Radiotherapy quality assurance in the PRO-GLIO trial: results from a dummy run comparing experts across twelve institutions in two Scandinavian countries
Source: Clin Transl Radiat Oncol. 2026 Jun 18;60:101220. doi: 10.1016/j.ctro.2026.101220 (PMC13316294; doi:10.1016/j.ctro.2026.101220)
Supplement: Supplementary material 11 — Treatment plan evaluation of target volumes with median values (range) [file mmc11.docx]

Supplementary Table 5: Treatment plan evaluation of target volumes with median values (range)

|  | Case 1 VMAT | | Case 1 PBT | | Case 2 VMAT | | Case 2 PBT | |
| --- | --- | --- | --- | --- | --- | --- | --- | --- |
| Volume | CTV | PTV | CTV | PTV | CTV | PTV | CTV | PTV |
| Homogeneity index | 0.04 (0.03-0.06) | 0.06 (0.04-0.08) | 0.03 (0.03-0.05) | 0.06 (0.05-0.11) | 0.05 (0.03-0.06) | 0.06 (0.05-0.14) | 0.04 (0.03-0.05) | 0.08 (0.05-0.12) |
| RTOG conformity index | 1.39 (1.29-1.49) | 1.08 (0.98-1.14) | 1.37 (1.20-1.47) | 1.06 (0.93-1.14) | 1.35 (1.24-1.41) | 1.07 (0.98-1.12) | 1.27 (1.23-1.43) | 1.00 (0.97-1.14) |
| Paddick/Van’t Riet conformity number | 0.72 (0.68-0.78) | 0.92 (0.87-0.96) | 0.73 (0.68-0.83) | 0.92 (0.87-0.95) | 0.74 (0.71-0.80) | 0.92 (0.89-0.96) | 0.78 (0.70-0.81) | 0.94 (0.88-0.95) |
| Lesion coverage volume factor | 1.00 (1.00-1.00) | 1.00 (0.97-1.00) | 1.00 (1.00-1.00) | 0.98 (0.92-1.00) | 1.00 (0.99-1.00) | 0.99 (0.94-1.00) | 1.00 (0.99-1.00) | 0.98 (0.95-1.00) |
| Healthy tissues conformity index | 0.72 (0.68-0.78) | 0.93 (0.87-0.99) | 0.73 (0.68-0.83) | 0.94 (0.87-0.99) | 0.74 (0.71-0.80) | 0.93 (0.89-0.97) | 0.79 (0.70-0.81) | 0.97 (0.88-0.98) |
| CTV: clinical target volume; PBT: proton beam therapy; PTV: planning target volume; RTOG: Radiation Therapy Oncology Group; VMAT: volumetric modulated arc therapy | | | | | | | | |
