# Supplementary material for: Radiotherapy quality assurance in the PRO-GLIO trial: results from a dummy run comparing experts across twelve institutions in two Scandinavian countries
Source: Clin Transl Radiat Oncol. 2026 Jun 18;60:101220. doi: 10.1016/j.ctro.2026.101220 (PMC13316294; doi:10.1016/j.ctro.2026.101220)
Supplement: Supplementary material 12 — Median dose (range) to organs of interest [file mmc12.docx]

Supplementary Table 6: Median dose (range) to organs of interest

| **Organ** | **Dose constraint** | **Case 1 VMAT** | **Case 1 PBT** | **Case 2 VMAT** | **Case 2 PBT** |
| --- | --- | --- | --- | --- | --- |
| Brain-CTV | V_30Gy_ ≤ 50% | 20.2% (14.0-27.8) | 15.3% (11.1-25.1) | 37.5% (30.0-42.7) | 27.1% (24.9-35.0) |
| Brain-CTV | D_Mean_ | 19.8 (16.6-22.0) | 9.3 (8.2-13.8) | 23.9 (22.2-24.9) | 17.0 (15.7-20.3) |
| Brainstem | D_0.03cc_ ≤ 54 Gy(RBE) | 53.6 (52.8-55.2) | 53.8 (51.9-54.8) | 53.2 (48.5-53.8) | 52.9 (51.5-54.3) |
| Retina, right | D_0.03cc_ ≤ 45 Gy(RBE) | 10.5 (7.4-14.2) | 0.2 (0.0-1.1) | 24.0 (12.5-31.1) | 21.3 (16.0-31.5) |
| Retina, left | D_0.03cc_ ≤ 45 Gy(RBE) | 22.4 (10.7-29.5) | 12.1 (4.1-16.2) | 19.0 (11.5-28.7) | 16.2 (5.8-19.9) |
| Hippocampus, right | D_40%_≤ 7,3 Gy(RBE) | 6.0 (3.5-7.0) | 0.0 (0.0-0.3) | 9.7 (7.5-32.2) | 5.9 (4.7-10.1) |
| Hippocampus, left | D_40%_≤ 7,3 Gy(RBE) | 53.8 (52.5-54.4) | 53.9 (52.5-54.1) | 8.5 (6.8-16.9) | 4.8 (2.2-10.9) |
| Cornea, right | D_0.03cc_ ≤ 30 Gy(RBE) | 7.8 (5.9-11.2) | 0.0 (0.0-0.8) | 13.3 (7.7-24.1) | 4.2 (0.8-13.1) |
| Cornea, left | D_0.03cc_ ≤ 30 Gy(RBE) | 10.6 (6.9-18.5) | 0.7 (0.0-3.0) | 13.1 (7.8-24.7) | 1.8 (0.4-8.1) |
| Optic nerve, right | D_0.03cc_ ≤ 55 Gy(RBE) | 22.1 (10.0-32.0) | 9.1 (2.3-17.6) | 53.4 (51.1-54.3) | 52.6 (52.2-53.9) |
| Optic nerve, left | D_0.03cc_ ≤ 55 Gy(RBE) | 52.6 (50.2-53.8) | 51.7 (48.7-53.5) | 53.3 (52.0-53.5) | 52.7 (51.9-53.8) |
| Lacrimal gland, right | D_Mean_ ≤ 25 Gy(RBE) | 8.5 (2.6-12.9) | 0.0 (0.0-0.0) | 14.7 (7.1-19.4) | 7.1 (3.5-10.5) |
| Lacrimal gland, left | D_Mean_ ≤ 25 Gy(RBE) | 19.3 (9.8-23.0) | 8.3 (1.5-17.1) | 9.8 (5.8-14.0) | 3.9 (1.9-12.3) |
| Optic chiasm | D_0.03cc_ ≤ 55 Gy(RBE) | 50.0 (42.4-54.7) | 50.1 (44.0-54.8) | 53.7 (53.0-54.4) | 53.8 (52.5-54.4) |
| Hypothalamus, right | D_Mean_ ≤ 45 Gy(RBE) | 25.7 (16.4-30.6) | 15.7 (4.3-29.4) | 52.4 (47.8-53.2) | 52.9 (49.1-54.1) |
| Hypothalamus, left | D_Mean_ ≤ 45 Gy(RBE) | 39.7 (31.9-44.3) | 29.4 (15.4-47.5) | 52.5 (49.3-53.3) | 53.0 (49.1-54.1) |
| Pituitary gland | D_Mean_ ≤ 20 Gy(RBE) | 24.1 (9.3-36.7) | 25.7 (5.9-31.3) | 21.1 (13.6-26.9) | 25.5 (4.3-33.8) |
| Lens, right | D_0.03cc_ ≤ 10 Gy(RBE) | 4.7 (3.4-7.7) | 0.0 (0.0-0.4) | 5.5 (3.8-7.0) | 0.6 (0.3-4.2) |
| Lens, left | D_0.03cc_ ≤ 10 Gy(RBE) | 6.4 (4.0-7.7) | 0.3 (0.0-1.2) | 5.0 (2.5-6.8) | 0.3 (0.0-2.3) |
| Cochlea, right | D_Mean_ ≤ 45 Gy(RBE) | 8.0 (3.1-11.4) | 0.1 (0.0-0.4) | 2.3 (1.8-7.0) | 0.1 (0.0-0.3) |
| Cochlea, left | D_Mean_ ≤ 45 Gy(RBE) | 29.8 (17.3-38.4) | 25.9 (10.4-38.9) | 2.5 (1.7-9.0) | 0.1 (0.0-2.3) |
| cc: cubic centimeters; CTV: clinical target volume; D_0.03cc_: dose to 0.03 cubic centimeters (near maximum dose); D_40%_: the minimum dose received by 40% of the volume; D_mean_: mean dose; Gy: Gray; PBT: proton beam therapy; RBE: relative biological effectiveness; V_30Gy_: volume receiving 30 Gy; VMAT: volumetric modulated arc therapy | | | | | |
