# Supplementary material for: Radiotherapy quality assurance in the PRO-GLIO trial: results from a dummy run comparing experts across twelve institutions in two Scandinavian countries
Source: Clin Transl Radiat Oncol. 2026 Jun 18;60:101220. doi: 10.1016/j.ctro.2026.101220 (PMC13316294; doi:10.1016/j.ctro.2026.101220)
Supplement: Supplementary material 13 — Differences in dose delivered to organs of interest (OOI) and target volumes were evaluated using the same treatment plan applied to all study centers’ delineations. Median dose (range) across study centers for OOI is reported, based on VMAT and PBT treatment plans developed at Oslo University Hospital for both cases. [file mmc13.docx]

Supplementary Table 7: Differences in dose delivered to organs of interest (OOI) and target volumes were evaluated using the same treatment plan applied to all study centers’ delineations. Median dose (range) across study centers for OOI is reported, based on VMAT and PBT treatment plans developed at Oslo University Hospital for both cases.

| **Organ** | **Dose constraint** | **Case 1 VMAT** | **Case 1 PBT** | **Case 2 VMAT** | **Case 2 PBT** |
| --- | --- | --- | --- | --- | --- |
| Brain-CTV | V_30Gy_ ≤ 50% | 23.1% (21.7-25.7) | 16.2% (11.1-25.1) | 44.0% (41.8-48.6) | 28.8% (25.9-34.6) |
| Brain-CTV | D_Mean_ | 22.5 (21.9-23.6) | 10.0 (9.2-10.0-11.5) | 25.9 (24.4-25.5) | 17.5 (15.9-27.8) |
| Brainstem | D_0.03cc_ ≤ 54 Gy(RBE) | 52.9 (51.5-54.6) | 51.6 (50.1-54.1) | 55.6 (50.2-56.5) | 54.9 (50.0-56.0) |
| Retina, right | D_0.03cc_ ≤ 45 Gy(RBE) | 12.6 (10.7-14.1) | 0.0 (0.0-0.0) | 28.2 (23.1-31.1) | 27.1 (25.5-29.5) |
| Retina, left | D_0.03cc_ ≤ 45 Gy(RBE) | 29.2 (26.1-30.3) | 9.0 (7.7-10.2) | 20.2 (17.5-21.0) | 17.7 (15.2-19.8) |
| Hippocampus, right | D_40%_≤ 7,3 Gy(RBE) | 7.1 (6.4-7.3) | 0.0 (0.0-0.0) | 7.9 (7.5-12.2) | 5.5 (4.4-11.5) |
| Hippocampus, left | D_40%_≤ 7,3 Gy(RBE) | 54.1 (53.6-54.5) | 52.9 (52.4-53.7) | 8.0 (6.9-9.1) | 2.8 (2.4-4.5) |
| Cornea, right | D_0.03cc_ ≤ 30 Gy(RBE) | 9.3 (6.4-9.8) | 0.0 (0.0-0.0) | 5.8 (4.0-7.3) | 1.2 (0.4-3.5) |
| Cornea, left | D_0.03cc_ ≤ 30 Gy(RBE) | 17.9 (17.0-23.5) | 0.3 (0.2-0.6) | 7.4 (4.2-10.7) | 0.4 (0.2-1.5) |
| Optic nerve, right | D_0.03cc_ ≤ 55 Gy(RBE) | 31.3 (29.1-32.1) | 8.2 (0.9-17.6) | 53.6 (51.7-53.9) | 52.7 (51.6-53.0) |
| Optic nerve, left | D_0.03cc_ ≤ 55 Gy(RBE) | 52.2 (49.9-54.1) | 52.0 (50.1-53.5) | 52.0 (41.7-52.4) | 51.9 (43.5-53.7) |
| Lacrimal gland, right | D_Mean_ ≤ 25 Gy(RBE) | 10.5 (5.6-11.6) | 0.0 (0.0-0.0) | 19.5 (7.0-28.6) | 10.3 (2.1-15.6) |
| Lacrimal gland, left | D_Mean_ ≤ 25 Gy(RBE) | 22.5 (18.8-23.5) | 2.7 (0.1-3.7) | 13.9 (3.8-18.8) | 5.7 (0.5-8.7) |
| Optic chiasm | D_0.03cc_ ≤ 55 Gy(RBE) | 51.8 (48.9-53.4) | 51.5 (48.8-53.3) | 53.7 (53.3-55.7) | 53.0 (52.0-54.8) |
| Hypothalamus, right | D_Mean_ ≤ 45 Gy(RBE) | 31.9 (29.3-32.0) | 19.3 (18.2-23.0) | 52.9 (49.5-54.2) | 53.0 (50.9-53.8) |
| Hypothalamus, left | D_Mean_ ≤ 45 Gy(RBE) | 43.1 (39.4-46.6) | 44.0 (38.9-47.4) | 52.2 (49.9-54.1) | 52.0 (49.9-53.7) |
| Pituitary gland | D_Mean_ ≤ 20 Gy(RBE) | 36.3 (35.4-37.1) | 27.5 (24.6-31.3) | 23.3 (17.3-38.1) | 24.9 (18.8-37.5) |
| Lens, right | D_0.03cc_ ≤ 10 Gy(RBE) | 3.8 (3.7-3.9) | 0.0 (0.0-0.0) | 3.7 (3.5-3.8) | 0.4 (0.3-0.5) |
| Lens, left | D_0.03cc_ ≤ 10 Gy(RBE) | 6.7 (5.9-6.9) | 0.1 (0.0-0.1) | 3.5 (3.4-3.7) | 0.2 (0.1-0.2) |
| Cochlea, right | D_Mean_ ≤ 45 Gy(RBE) | 11.2 (11.0-11.5) | 0.0 (0.0-0.0) | 1.8 (1.8-1.9) | 0.0 (0.0-0.0) |
| Cochlea, left | D_Mean_ ≤ 45 Gy(RBE) | 36.4 (33.8-37.2) | 35.0 (32.5-36.0) | 1.7 (1.7-1.8) | 0.0 (0.0-0.0) |
| GTV | D_99%_ ≥ 51.30 Gy(RBE) | 52.7 (46.0-52.7) | 52.0 (43.3-52.1) | 52.2 (52.1-52.3) | 52.7 (52.2-52.8) |
| CTV | D_99%_ ≥ 51.30 Gy(RBE) | 52.8 (35.2-52.97) | 52.2 (23.8-52.5) | 52.4 (46.2-52.5) | 51.9 (46.5-52.5) |
| cc: cubic centimeters; CTV: clinical target volume; D_0.03cc_: dose to 0.03 cubic centimeters (near maximum dose); D_40%_: the minimum dose received by 40% of the volume; D_99%_: the minimum dose received by 99% of the volume; D_mean_: mean dose; Gy: Gray; PBT: proton beam therapy; RBE: relative biological effectiveness; V_30Gy_: volume receiving 30 Gy; VMAT: volumetric modulated arc therapy | | | | | |
